# Haptic Feedback-Based Virtual Reality Intervention for a Child With Infantile Hemiplegia: A Case Report

**DOI:** 10.7759/cureus.23489

**Published:** 2022-03-25

**Authors:** Chanan Goyal, Vishnu Vardhan, Waqar M Naqvi

**Affiliations:** 1 Paediatric Physiotherapy, Government Physiotherapy College, Raipur, IND; 2 Physiotherapy, Datta Meghe Institute of Medical Sciences, Wardha, IND; 3 Research, NKP Salve Institute of Medical Sciences & Research Centre, Nagpur, IND

**Keywords:** playstation, case report, hand function, upper extremity function, functional independence, haptic feedback, virtual reality, hemiparesis, hemiplegia, infantile hemiplegia

## Abstract

Virtual reality (VR) refers to an advanced technology that provides real life-like experience in a virtual environment. Numerous commercially available systems provide gaming opportunities with VR, while a few also provide haptic feedback along with VR. In the recent past, VR has been explored as a viable intervention in the field of neurorehabilitation. Although there are promising results for adults with hemiplegia, the research involving children with infantile hemiplegia is in the nascent stage. Infantile hemiplegia is manifested by sensory and motor deficits predominantly on one side of the body resulting in adverse effects on the functionality of the affected side since early life. VR gaming has an intense, motivational component that encourages children to put sustained voluntary effort to use both upper extremities. A six-year-old male with infantile hemiplegia presented with difficulty in using the left upper extremity. Pre-intervention scores of the nine-hole peg test (9HPT) and box and block test (BBT) were used to evaluate the manual dexterity, while those of ABILHAND-kids and functional independence measure for children (WeeFIM self-care section) assessed the functional independence. The child underwent treatment for six weeks (five days/week), with each session lasting for 60 minutes/day that included VR gaming with haptic feedback for 30 minutes and conventional physiotherapy for 30 minutes. Post-intervention scores were recorded and were compared with pre-intervention scores. Marked improvement in left upper extremity function was noted not only objectively by the outcome measures but also subjectively by the parents as well as by the child. Moreover, the child remarked that he enjoyed the therapy sessions. The findings of this report would facilitate the design of further research in this area in the form of larger trials.

## Introduction

Infantile hemiplegia refers to weakness of one side of the body since early life. More than half of the children with infantile hemiplegia have more impairment in the upper extremity than lower extremity [1]. This ultimately leads to an increase in the burden of care on the caregivers.

Virtual reality (VR) with haptic feedback refers to an advanced technology that provides a real-life-like experience in a virtual environment in which a gamer responds not only to audio-visual feedback but also to force feedback. It has been explored as a viable intervention in the field of neurorehabilitation in the recent past. Although there are promising results for adults with hemiplegia [2,3], the research involving children with infantile hemiplegia needs impetus.

In a study conducted by Ravi et al., it was concluded that further investigation is required for establishing VR gaming as an evidence-based intervention in the neurorehabilitation of children [4]. Haptic feedback along with audio-visual feedback facilitates adaptive motor response [5]. VR gaming has an intense, motivational component that promotes sustained voluntary effort to use both upper extremities by the child. This motivation was tapped to improve hand function in the case presented here. This report aimed to utilise the appeal in VR gaming system and haptic feedback for bringing out the child’s inherent potential to use his affected upper extremity and objectively record its’ effectiveness in improving function.

## Case presentation

Patient information

A six-year-old male child presented with left side weakness of the body. Parents had a major concern that their child is reluctant to use his left hand. As per his parents, he was born full-term by normal delivery, but he had a delayed birth cry. They noticed his preferential hand usage of his right hand on his first birthday. Activities like reaching out, grasping, carrying, and releasing by the left upper limb were inadequate. He required assistance in activities of daily living. Parents mentioned that the child needed repeated prompts for the use of paretic hand and that he refrained from taking the initiative to put the affected hand to function. He started walking independently at the age of 18 months but with a raised left heel. At the age of four years, a paediatric neurologist was consulted, who made a clinical diagnosis of infantile hemiplegia and referred the child to a paediatric physiotherapist. Due to the COVID-19 pandemic situation, the parents could take the child to consult a paediatric physiotherapist after two years when the child was six years old.

Clinical findings

On examination, the child had hypertonicity in the left upper limb and lower limb. There was tightness in left elbow flexors, forearm pronators, wrist and finger flexors. He usually kept his left hand fisted with his thumb in his palm. Besides, he displayed mild tightness of the left calf muscle and walked with a slightly raised heel on the left side. Weakness in elbow extensors, forearm supinators, wrist extensors, finger extensors, thumb abductors, and ankle dorsiflexors was noted. He was on a level I on Gross Motor Function Classification System (GMFCS) but level III on Manual Ability Classification System (MACS). He used his right upper extremity for tasks that required one hand, but he sought help for bimanual tasks. He did not try to use his left upper extremity even on request. He was able to understand commands and communicate age appropriately.

Baseline data was collected using objective outcome measures. He took 58 seconds to put all pegs by left hand during the nine-hole peg test (9HPT). In 60 seconds, he could transfer 15 blocks from one side of the partition to the other during the box and blocks test (BBT). ABILHAND-kids score was 24, and Rasch analysis showed 0.951 logits (57% of logits) with a standard error of 0.443 logits (3% of logits). His score in the self-care section of the Functional Independence Measure for children (WeeFIM) was 31.

Therapeutic intervention

After an initial assessment, the ankle-foot orthosis was prescribed, and attending regular physiotherapy sessions on weekdays was recommended.

**Figure 1:**
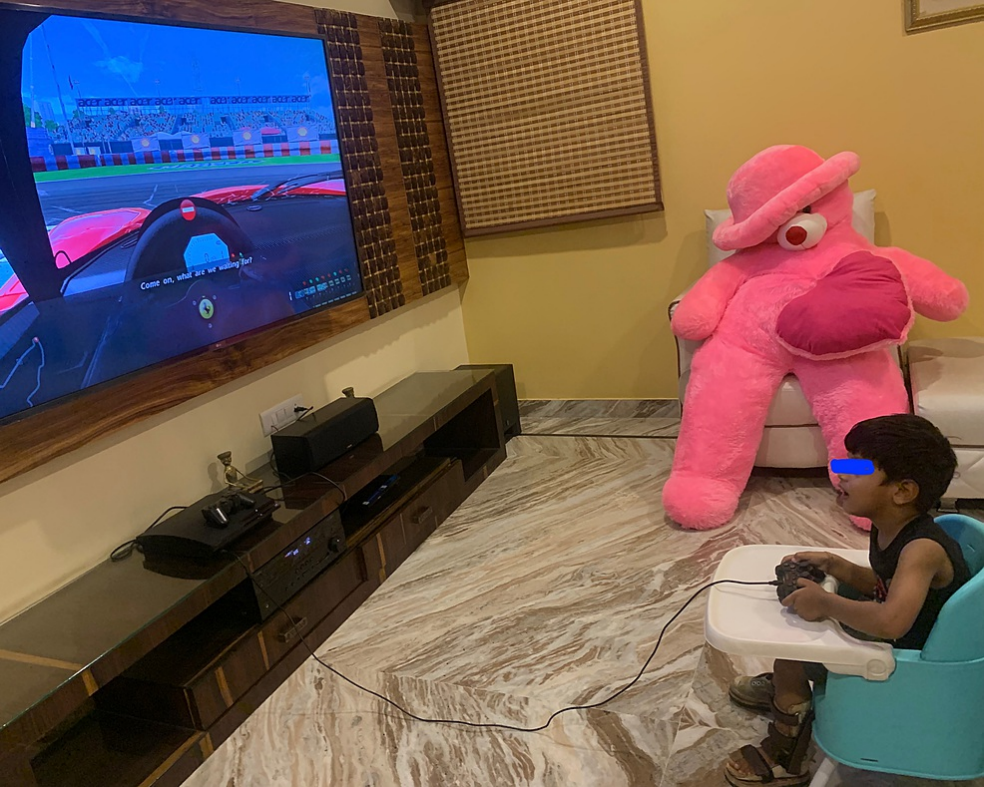
The child with infantile hemiplegia playing a haptic feedback enhanced VR game using PlayStation

Each session lasted for 60 minutes. The child underwent 30 minutes of conventional physiotherapy, including activities for strengthening weak muscles like elbow extensors, forearm supinators, wrist extensors, finger extensors, and ankle dorsiflexors. Stretching of tight muscles like elbow flexors, forearm pronators, wrist flexors, finger flexors, and ankle plantar flexors was done. Weight-bearing activities like push-ups and quadruped positions were imparted. This was followed by 30 minutes of VR and haptic feedback-based training of upper extremity by playing a serious game with a real-life-like experience. PlayStation 4 (Sony Interactive Entertainment Inc., Minato, Tokyo, Japan) was used as a VR system. Haptic feedback was provided by the hand control unit and a car racing game ‘Gran Turismo - The real driving simulator’ was played by the child. He could see the avatar of his hands controlling the steering wheel on the television screen. He held the remote-control unit with both hands and actively abducted his left thumb to press the buttons, which he rarely attempted otherwise. He was highly engaged in the game and handled the controls well. The training was carried out for six weeks (five days a week). Figure 1 shows the child playing the VR base car-racing game using a remote control that provides haptic feedback.

Follow-up and outcomes

Post-intervention, he took 39 seconds to put all pegs by left hand during 9HPT showing an improvement of 48.7% in fine motor dexterity. In 60 seconds, he could transfer 26 blocks from one side of the partition to the other during BBT, which showed a 73.3% improvement in gross motor dexterity. ABILHAND-kids score was 29, and Rasch analysis showed 1.984 logits (65% of logits) with 0.481 logits (4% of logits), indicating an improvement of 8% in the manual ability to perform day-to-day tasks. His score in the self-care section of the WeeFIM was 37 revealing an increase of 19.35% in functional independence and reduction in the burden of care on the parents.

## Discussion

According to a few previous studies, VR has emerged as a promising intervention for improving motor function in children with neuromotor disorders [4,6-9]. Infantile hemiplegia results in diminished motor control of the affected upper extremity leading to the neglect and learned disuse by the child that hampers its sensory-motor development [10]. The vicious cycle of weakness and disuse needs to be broken for improving function, which is usually a challenging task for a child with infantile hemiplegia who is otherwise ambulatory. Though constraint-induced movement therapy has been considered effective in the management of infantile hemiplegia, it lacks the much-needed motivation for the child that limits the number of repetitions of movements and the long-term compliance [11]. According to a previous study, PlayStation is a motivational training tool and has the potential to improve upper extremity (UE) function in children [12]. In a recent study published in 2020, eye-hand coordination was found to be improved by VR-based therapy in children [13].

In the presented case, 9HPT was used to evaluate fine motor function. The child displayed marked improvement in this domain that can be attributed to the repetitive practice of fine manipulation of the buttons on the handheld controls while playing VR games with haptic feedback during the intervention. Smith et al. demonstrated moderately high test-retest reliability and high inter-rater agreement of 9HPT [14]. Correlations between the scores on the 9HPT and Purdue Pegboard Test revealed sufficient concurrent validity of these measures, and a significant difference in test scores between regular and special education groups provided further indication of construct validity. Thus, 9HPT is considered to be an effective tool for estimating the fine motor dexterity of school-aged children [14].

Out of all the outcome measures, the child displayed maximum improvement in the score of BBT, which can be attributed to the practice of grasping remote control while playing VR games. BBT was used as a test of unilateral gross manual dexterity. It is administered by asking the subject to one-by-one move the maximum number of blocks from one compartment of a box to another of equal size within 60 seconds. The test has shown very high inter-rater, test-retest reliability, and excellent construct validity [15].

ABILHAND-kids was used to evaluate upper extremity function, which is a 21-item functional ordinal scale specifically developed to measure manual ability. Its measurement precision is considered highly adequate for clinical practice. Parents are requested to fill out the questionnaire based on their perception of the level of difficulty that a child faces during the performance of each activity in the list on a three-tier scale of ‘Impossible’, ‘Difficult’, ‘Easy’ [16]. The linear measure obtained by the Rasch model can be used to make a comparison quantitatively regarding the manual ability of a child [17]. The test has shown excellent test-retest reliability as well as internal consistency [16]. It has excellent convergent validity with MACS [18]. In the presented case, the positive change in the score of this test indicated the carry-over effect of the intervention on functional activities.

WeeFIM (self-care section) was used for evaluating functional independence. Functional independence measure for children is also known as WeeFIM. It can be used to estimate the ‘burden of care’ for children with developmental disabilities between 6 months to 21 years of age [19]. The performance of the task is categorised as ‘dependent’ if the score is between 1-5 and is categorised as ‘independent’ if the score is six or seven. Score one indicates total assistance, score two indicates maximal assistance, score three indicates moderate assistance, four indicates minimal assistance, score five indicates supervision or set-up, score six indicates modified independence that includes an assistive device usage or not completing the task in a timely or safe manner and score seven indicates complete independence that is the child completes the task without using a device [19]. The test-retest reliability is excellent for children with disabilities [20]. The recorded change in the score of this test in the presented case point towards the enhancement in functional independence of the child.

In the present study, the child remarked that he looked forward to the enjoyable sessions. He reported that his control over the left extremity, especially the thumb, improved at the end of six weeks of intervention. Apart from conventional physiotherapy, this can be attributed to the VR-based rehabilitation that was characterised by repetitive task-specific movements that included abduction and opposition of the thumb of the affected side, which was difficult for the child previously. Haptic feedback must have helped him to finely tune the motor response within a virtual environment. Better eye-hand coordination must be one of the contributing factors in improved performance. In addition, after the intervention, his parents perceived that he required fewer verbal prompts in bimanual tasks and that he was less dependent on them for his routine activities.

## Conclusions

The child not only showed improvement in his hand function but the burden of care on the caregivers was also decreased. The improvement in upper extremity function was more than the improvement in functional independence of the child. Moreover, the parents and the child reported subjectively that his left-hand use noticeably increased in activities of daily living. Thus, the use of VR games with haptic feedback seems to be an engaging and cost-effective intervention in such cases. Besides, further investigation is warranted to explore and enhance the carry-over effect of this intervention on function and participation. The findings of this study pave the way for future research that will help to gain a better understanding of the utility of haptic feedback enhanced VR-based physiotherapy intervention for children with infantile hemiplegia.
